# Natural Antisense Transcripts and Long Non-Coding RNA in *Neurospora crassa*

**DOI:** 10.1371/journal.pone.0091353

**Published:** 2014-03-12

**Authors:** Yamini Arthanari, Christian Heintzen, Sam Griffiths-Jones, Susan K. Crosthwaite

**Affiliations:** The Michael Smith Building, Faculty of Life Sciences, University of Manchester, Manchester, United Kingdom; University of Missouri, United States of America

## Abstract

The prevalence of long non-coding RNAs (lncRNA) and natural antisense transcripts (NATs) has been reported in a variety of organisms. While a consensus has yet to be reached on their global importance, an increasing number of examples have been shown to be functional, regulating gene expression at the transcriptional and post-transcriptional level. Here, we use RNA sequencing data from the ABI SOLiD platform to identify lncRNA and NATs obtained from samples of the filamentous fungus *Neurospora crassa* grown under different light and temperature conditions. We identify 939 novel lncRNAs, of which 477 are antisense to annotated genes. Across the whole dataset, the extent of overlap between sense and antisense transcripts is large: 371 sense/antisense transcripts are complementary over 500 nts or more and 236 overlap by more than 1000 nts. Most prevalent are 3′ end overlaps between convergently transcribed sense/antisense pairs, but examples of divergently transcribed pairs and nested transcripts are also present. We confirm the expression of a subset of sense/antisense transcript pairs by qPCR. We examine the size, types of overlap and expression levels under the different environmental stimuli of light and temperature, and identify 11 lncRNAs that are up-regulated in response to light. We also find differences in transcript length and the position of introns between protein-coding transcripts that have antisense expression and transcripts with no antisense expression. These results demonstrate the ability of *N. crassa* lncRNAs and NATs to be regulated by different environmental stimuli and provide the scope for further investigation into the function of NATs.

## Introduction

High-throughput sequencing has revealed that the overwhelming majority of the eukaryotic genome is transcribed. For example, the ENCODE project has annotated transcription originating from around three quarters of the human genome [1], [2], [3]. Similarly, the majority of the mouse genome has also been shown to be transcribed [4]. Novel transcribed regions may represent extensions of known protein-coding genes, novel protein-coding transcripts, and transcripts that do not appear to have protein-coding capacity [5]. An ever-increasing number of classes of non-protein-coding RNAs have been discovered and annotated, from the well-known housekeeping small RNAs (including ribosomal RNA and transfer RNA), and regulatory small RNAs (such as small interfering RNA, microRNAs, and piwi-associated RNAs), to long non-coding RNAs (lncRNAs, or lincRNAs; reviewed in [6] and [7]). In particular, high-throughput technologies have highlighted thousands of lncRNAs in a range of eukaryotic organisms, from yeast to humans [8], [9], [10], [11]. A handful of examples have been well-characterised and shown to have roles in the transcriptional and post-transcriptional control of gene expression via RNA-protein, RNA-DNA and RNA-RNA interactions. For instance, lncRNAs have been shown to be involved in chromatin modification, cell fate determination, and × chromosome inactivation (reviewed in [7], [12]). However, the function of the vast majority of lncRNAs remains a mystery.

A subset of long non-coding RNAs is a class of so-called Natural Antisense Transcripts (NATs), containing transcripts with sequence complementarity to other RNAs. NATs can be divided into *cis-* and *trans*-NATs. *Cis*-NATs arise from the same genomic region as their complementary sense transcript, whereas *trans*-NATs are complementary to transcripts from remote loci. Specific antisense transcripts have been shown to regulate the expression of their sense transcripts via a range of mechanisms including: inhibition of transcription due to steric clashes of the transcriptional machinery; repression of expression due to competition for transcription factors; silencing the expression of the sense protein by RNAi; disruption of post-transcriptional modification and translation of the sense transcript by forming RNA/RNA duplexes; and masking of specific signals on the sense RNA necessary for splicing, stability or degradation (reviewed in [13], [14] and [15]). In eukaryotes, antisense transcripts have been found to be prevalent in the human genome [16], other mammalian genomes [17], plant [18], and fungal genomes (reviewed in [19]).

Fungi provide a simple eukaryotic model in which to understand important and widespread mechanisms of gene regulation. The few genome-wide searches for antisense transcription in fungi have indicated that *cis*-NATs are expressed from the opposite genomic strand of 15-50% of protein-coding loci. In *Saccharomyces cerevisiae*, antisense transcripts associated with more than a thousand genes are expressed [20], [21], and evolutionarily conserved [21], [22], [23]. Genes with antisense expression overlapping the sense transcript at the 3′ UTR were more likely to involved in regulatory functions [20], whilst genes that had antisense transcripts spanning more than 75% of their length were found to be enriched for genes induced during stress, growth, meiosis and sporulation [21]. In *Schizosaccharomyces pombe*, 2409 protein-coding genes were found to have *cis*-NAT expression. Again, genes involved in meiosis were more likely to be associated with *cis*-NAT transcription [24]. The association of NATs with loci in certain ontology categories and their differential expression during development and in response to external stimuli suggests a regulatory role. In the pathogen *Aspergillus flavus*, differentially regulated NATs are found antisense to genes involved in temperature-sensitive morphogenesis and aflatoxin biosynthesis [25].

Of the filamentous fungi, resources for investigating gene function are most advanced in *Neurospora crassa*. Although *N. crassa* is non-pathogenic, it is closely related to a number of important animal and plant pathogens. The *N. crassa* genome has been fully sequenced and assembled [26] and a wide range of molecular genetic tools are available [27], [28], [29]. *Neurospora* displays complex cellular and morphological organisation [30], and has been utilised as a model for the study of numerous cell and developmental phenomena including the circadian clock, RNAi, and sexual and asexual development [31]. Moreover, one of the few well-characterised fungal non-coding NATs, qrf, was described in *Neurospora* [32], [33]. *qrf* is a lncRNA transcribed antisense to the circadian clock gene, *frequency* (*frq*) [34]. *qrf* expression affects the clock's response to light [32] *via* chromatin modification at the *frq* promoter [33]. The only previous genome-wide analysis of antisense transcription in *N. crassa* predicted the presence of 87 pairs of sense/antisense ORFs using computational methods [35].

Here, we annotate a total of 939 novel lncRNAs in *N. crassa* using RNAseq from cultures grown in the dark and under conditions of light and temperature stimulation. 477 of our lncRNAs are antisense to annotated protein-coding genes, and we find 38 novel pairs of sense/antisense lncRNAs. We have also characterised protein-coding transcripts that are associated with NATs and determined their expression, NAT overlap, number and position of introns. We report the differential expression of lncRNAs in response to light and temperature using RNAseq data and confirm the expression of several sense/antisense transcript pairs by qPCR. The expression of these candidate sense/antisense pairs was observed in a *dicerlike-1, dicerlike-2* double mutant as well as in *upf1* mutant strains to determine if they were substrates for RNAi or nonsense mediated decay (NMD) pathways.

## Results

### Annotation of novel *Neurospora* transcripts by RNAseq

Using ABI SOLiD sequencing, we have sequenced the transcriptome of wild-type (*54–3, bd a*) and *wc-2* ^▵^, *vvd* ^▵^, *frq* ^▵^, *wc-1* ^▵^, *bd (quad^▵^*) strains of *N. crassa* under three conditions (dark, light pulse and temperature pulse; two biological replicates for each condition). The number of reads obtained for each dataset varied between 15 and 52 million reads. Using the splice-aware mapping tool, Tophat [36], 37 million reads from the WT and 50 million reads from the *quad^▵^* datasets mapped to unique locations in the NC10 version of the genome (see Table S1); all other reads were not considered further.

To annotate novel transcripts, all datasets of mapped reads were merged, and all reads separated by less than 200 nts on the same genomic strand were clustered, as described in the Methods section. This pipeline predicts 29,605 transcribed regions (termed transfrags here). 69.8% (6922 of 9907) of transcripts annotated in the BROAD *N. crassa* database are represented by transfrags with 50 or more mapped reads. In addition, we identify 3,765 transfrags that are represented by more than 50 reads in one of the combined datasets (WT or *quad^▵^*) and that do not overlap an annotated gene. However, 2,652 of these transfrags are located within 500 nts of the ends of annotated protein-coding genes on the same genomic strand, and may therefore represent unannotated terminal exons. Since 92% of annotated protein-coding genes are separated by more than 500 nts (Figure S1) the 2,652 transfrags lying within 500 nts of an annotated gene were discarded from subsequent analyses. This leaves 1,113 putative non-coding transcripts with > = 50 reads in either the WT or the *quad^▵^* dataset (Table S2).

**Figure 1:**
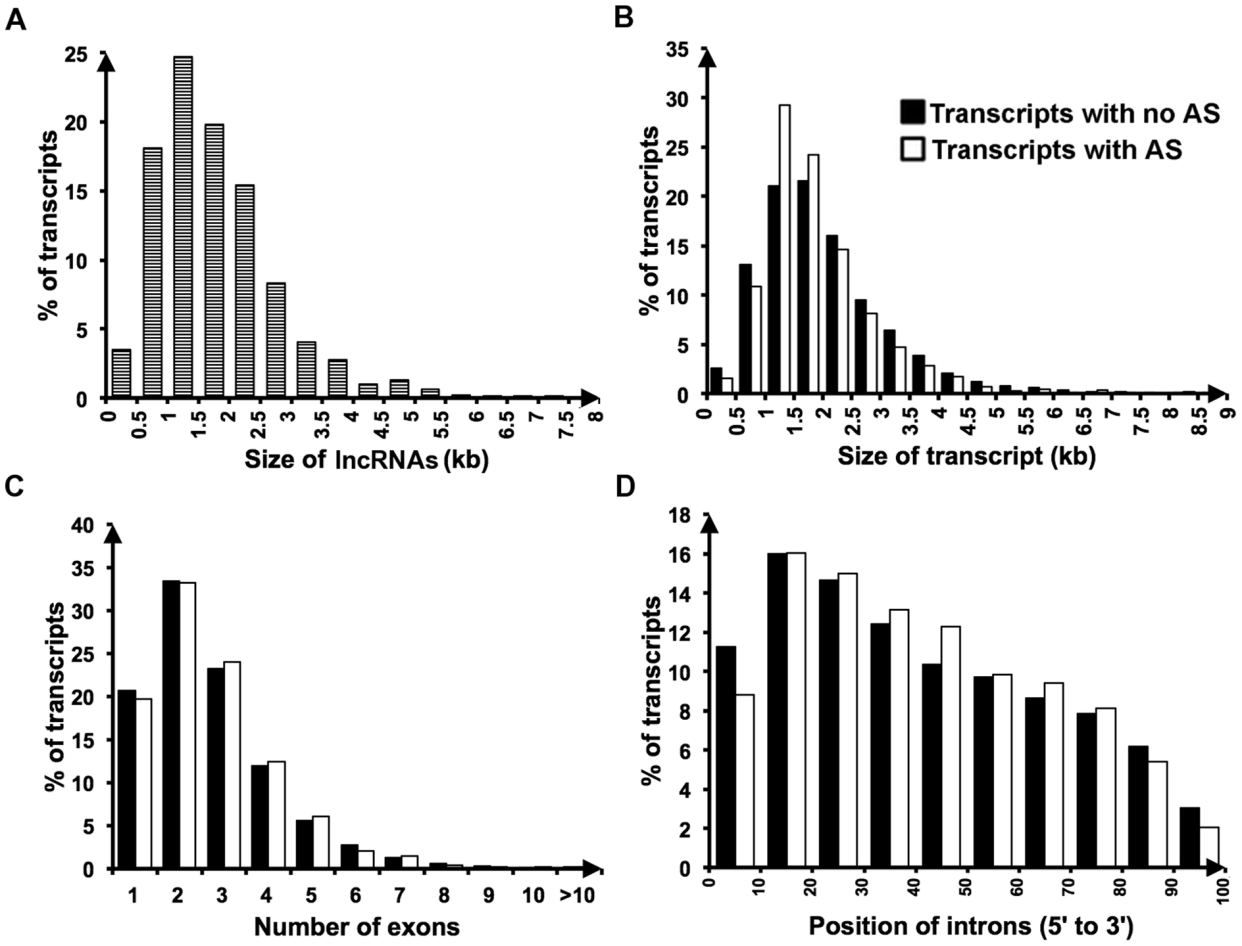
Properties of novel lncRNAs and protein-coding transcripts with and without antisense transcription. **A**. Size distribution of lncRNAs, **B**. size distribution, **C**. number of exons, and **D**. position of introns in annotated transcripts with (black bars) and without (white bars) antisense transcription.

To assess the coding potential of the 1,113 transcripts we used the Coding Potential Calculator (CPC) [37] as described in [38]. We first tested CPC on the dataset of all annotated protein-coding transcripts in the BROAD database [39]. CPC predicted over 96% (9,559 of 9,907) of the annotated genes to have protein-coding potential. Of the 348 annotated genes predicted to have no coding potential, only 10 are annotated as proteins of known function, 7 of which have an ORF of less than 100 amino acids. In contrast, CPC reported only 78 of our putative non-coding transcripts as having protein-coding potential (7%) (Table S2). The transcripts that were predicted to have coding potential were discarded. We further discarded 96 putative non-coding transcripts that have high-scoring matches to models of annotated non-coding RNA families from the Rfam database – the majority of these sequences are predicted tRNAs and snoRNAs. This left 939 long non-coding RNAs (lncRNA) (Table S2). A size distribution of our lncRNA set is shown in Figure 1A. In common with most other lncRNA sets, the sequences of our lncRNAs are not well-conserved in other fungal genomes. We find that only 9 of the 462 lncRNAs that are not antisense to annotated protein-coding transcripts display extended regions of sequence similarity in Sordariomycetes genomes outside of the *Neurospora* clade, of which only 2 are conserved in other Pezizomycotina, and none in more distant Ascomycetes (see Methods). Unsurprisingly, the large majority (427) of the sequences are conserved in the *Neurospora tetrasperma* genome (Table S2).

### Sense/antisense transcript pairs

In the BROAD *N. crassa* genome database [39], 428 sense/antisense transcript pairs that overlap by at least 1 nt are annotated (Table S3) and 357 pairs (83%) of these overlap by more than 25 nts. From the collection of annotated sense/antisense pairs, 324 pairs (76%) have evidence for expression of both sense and antisense transcripts (>50 reads) in our combined RNAseq datasets, and a further 92 transcripts have evidence for either the sense or antisense transcript. Almost all of the annotated sense/antisense transcript pairs (419; 98%) are convergently transcribed, with overlap of the 3′ ends of the transcripts, 9 pairs represent divergent genes with 5′ overlaps, and there is only one example of overlapping sense/antisense ORFs.

**Figure 2:**
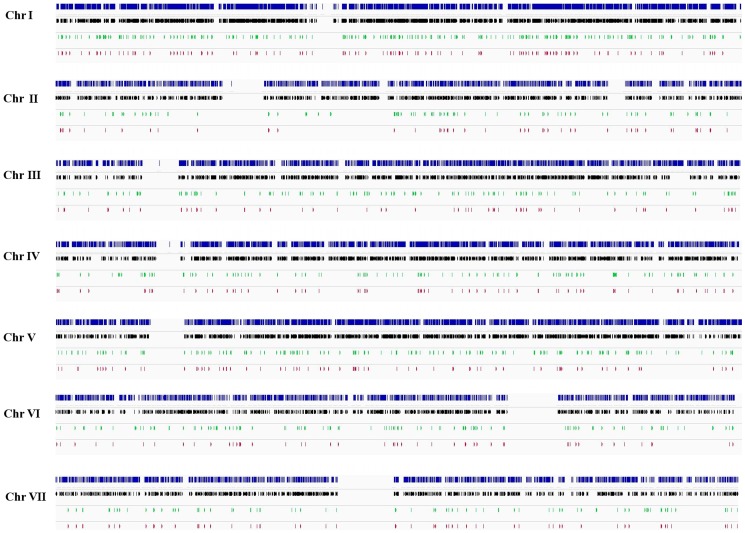
Genomic distribution of annotated and lncRNA transcripts. Annotated genes from the BROAD database are depicted in blue and those that are expressed above a threshold of > = 50 reads in our combined datasets are depicted in black. The distribution of all lncRNAs is shown in green and antisense transcripts are shown in red. Large gaps in the gene annotation indicate centromeric regions.

In our dataset, 513 annotated protein-coding transcripts, representing over 5% of all annotated genes, display evidence of antisense lncRNA transcription (> = 50 antisense reads). Eleven of these protein-coding transcripts have two antisense lncRNAs separated by more than 200 nts. One such pair represents the *qrf* transcript antisense to *frequency* (*frq*), for which the transcript boundaries are well-determined ([32] and Crosthwaite SK, unpublished). Hence, we combined the two fragmentary lncRNAs that are antisense to *frq* to form one single transcript. Conversely, there are 36 cases where a single lncRNA is antisense to multiple annotated transcripts, including 8 lncRNAs antisense to multiple isoforms of the same gene. In total, we annotate 477 lncRNAs antisense to 513 known genes (Table S4). The lncRNAs are uniformly distributed across all chromosomes (Figure 2; χ^2^ test *p*>0.05). To eliminate the possibility that the antisense lncRNAs could be annotated based on reads that are mapped to the incorrect genomic strand (for example, due to PCR errors in the library preparation), we examined the mapped reads for splice junctions. 315 of our antisense lncRNAs (66%) have consensus splice junctions (GT-AG, GC-AG and AT-AC) supported by reads spanning the intron. We identified 5 occurrences of the nonconsensus splice junction CT-AC, which could represent incorrectly orientated reads. However, the same predicted lncRNA transcript also contained other consensus splice sites supported by read data, indicating the correct orientation of the antisense transcript. The remaining 162 antisense transcripts have no evidence of introns.

The majority (258) of antisense lncRNAs are transcribed convergently with their protein-coding sense partner, such that their 3′ ends overlap. 63 sense/antisense transcripts are divergently transcribed and overlap at their 5′ ends. There are 43 cases of antisense lncRNAs nested within the bounds of the sense protein-coding gene, and 149 annotated sense genes nested within antisense lncRNAs. Almost 97% of our antisense lncRNAs (461) overlap the ORF of the annotated protein-coding transcripts. We also identify 38 pairs of sense/antisense lncRNAs originating from previously unannotated loci (Table S6). 36 of these pairs overlap by more than 200 nts, and we again observe an excess of convergently transcribed sense/antisense pairs (14 of these pairs overlap at the 3′ end, and 9 at the 5′ end).

We next assessed whether the transcripts with antisense expression (coding or non-coding) exhibit any particular characteristics. The lengths of transcripts with associated antisense RNAs are present at a greater proportion in the 1–2 kb size range, however there was no significant difference between the distributions of lengths between transcripts with and without antisense RNA (Figure 1B). Similarly there was no difference in the number of exons (Figure 1C). However, the distribution of intron positions is significantly different (*p*-value 0.001); transcripts with antisense expression have fewer introns at the 5′ and 3′ ends (Figure 1D). The transcripts with antisense expression were found to be enriched in a number of functional categories, including metabolism (extracellular polysaccharide degradation, extracellular metabolism, metabolism of lysine), extracellular/secretion protein, antiporter, and oxidation of fatty acids (*p*-value < = 0.05, FunCat [40]; Table S5).

Divergent transcripts may arise from bidirectional promoters. In our dataset, 355 lncRNAs, including 233 antisense lncRNAs, are located upstream (within 1 kb) of annotated genes on the opposite strand. Without detailed experimental characterisation of a promoter region, it is difficult to determine whether or not a bidirectional promoter is responsible for the expression of divergently transcribed transcripts. However, we find 2 examples, NCU07267/NCU07268_AS and NCU01107/NCU01106_AS, where the divergently transcribed gene and lncRNA are both significantly up-regulated in response to light (see below).

It has previously been suggested that the terminators of protein-coding genes could act as promoters for antisense transcripts [41]. Using the same criteria as Murray *et al*. [41], we find that 42% of antisense long non-coding transcripts have their start sites between 100 nts upstream and 600 nts downstream of the stop codon of protein-coding ORFs, and therefore may arise from terminator regions of sense genes. In order to avoid confusing potential terminator-derived antisense transcripts with transcripts arising from nearby bidirectional promoters, this analysis ignored all pairs of protein-coding genes that lie closer than 500 nts on the same strand.

### Differential expression of transcripts following light and temperature pulses

Our datasets further allowed us to identify lncRNAs under the direct or indirect control of light- or temperature. We used DESeq to determine the differential expression of transcripts between the control dark-grown culture at 25 °C and cultures exposed to light or 30 °C. Eleven lncRNA, were found to be up-regulated in response to light (5% FDR; see Table 1), of which 7 are antisense to protein-coding genes. None of the lncRNAs were found to be differentially expressed in response to temperature. Although some of the lncRNAs identified in this study overlap introns of their sense counterparts, we found no evidence of alternative splicing occurring in transcripts due to changes in expression of either sense or antisense RNA.

**Table 1:** List of lncRNAs differentially expressed in response to light.

	lncRNA*	Sense transcript	Mean normalized read count WT-Dark	Mean normalized read count WT-Light	Fold change	*p* value	*p*_adj_
1	NC_lncRNA_995		15.1	244.8	16.2	1.78×10^−18^	9.29×10^−16^
2	NCU07268T0_AS	Hypothetical protein	2.6	165.1	62.5	3.07×10^−11^	6.54×10^−9^
3	NCU01106T0_AS	*L-amino acid oxidase*	4.7	71.9	15.4	4.59×10^−8^	6.71×10^−6^
4	NCU05853T0_NCU05852T0_NCU05851T0_AS	*MFS sugar transporter, glucuronan lyase A, 4-coumarate-CoA ligase 1*	48.3	159.3	3.3	1.43×10^−5^	0.00135
5	NCU02265T0_AS_qrf	*frequency*	38.9	146.6	3.8	2.76×10^−5^	0.00239
6	NC_lncRNA_429		1.9	24.9	12.8	0.00012	0.009
7	NC_lncRNA_418		0	28.7	Inf	0.0001	0.009
8	NCU09039T0_AS	Hypothetical protein	6.6	44.9	6.8	0.0001	0.010
9	NCU09616T0_AS	Hypothetical protein	4.9	32.8	6.7	0.0003	0.021
10	NCU04252T0_AS	*SNARE docking complex subunit*	3.9	38.8	9.7	0.0004	0.025
11	NC_lncRNA_1037		32.8	151.7	4.6	0.0004	0.025

*Locus information can be found in Table S2.

### Verification of sense/antisense transcript expression using qPCR

**Figure 3:**
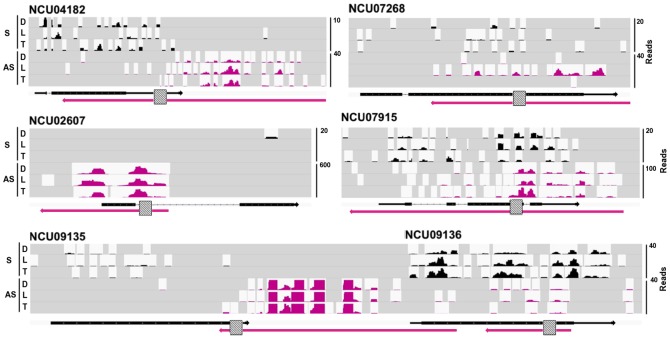
Examples of sense/antisense transcript pairs annotated from RNA sequencing data and validated by qPCR. Panels display the locations and distribution of RNAseq reads of sense protein-coding (black) and antisense lncRNA (pink) transcripts. RNAseq reads from the WT dark (D), light pulse (L) and temperature pulse (T) samples mapping to each locus are shown; read count scales differ. Below each panel, arrows represent sense (black) and antisense (pink) transcripts. Thick lines represent exons and thin lines introns. Grey boxes indicate the approximate region of each transcript amplified by qRTPCR. Reads are shown for the following sense transcripts and their complementary antisense RNAs: NCU04182 (coding for splicing factor 3 b subunit 4), NCU07268 (coding for a hypothetical protein with PAS domain), NCU02607 (coding for hypothetical protein), NCU07915 (coding for integral membrane protein), NCU09135 (coding for phosphatidylinositol phospholipase C) and NCU09136 (coding for a hypothetical protein. A single antisense transcript overlaps both NCU09135 and NCU09136. The two transcripts antisense to NCU09136 are separated by more than 200 nts.

**Figure 4:**
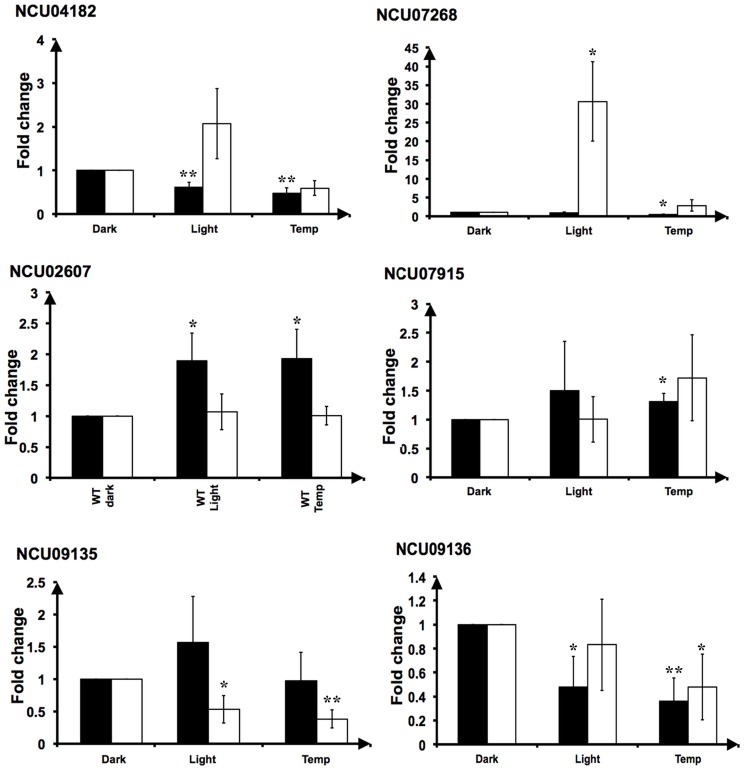
qPCR verification of expression of selected sense/antisense pairs. Expression of both the sense and antisense transcript for NCU04182, NCU07268, NCU02607, NCU07915, NCU09135 and NCU09136 in the WT is shown, after growth in the dark, and exposure to light and temperature pulses. Black bars indicate the protein-coding sense transcript and white bars indicate its antisense transcript. Error bars represent standard deviation. Statistical significance between light vs dark and temperature vs dark was determined using Student t test, * indicates *p*-value <0.05 and ** indicates *p*-value < = 0.005. n = 3.

We validated the expression of six pairs of sense and antisense transcripts (Figure 3) chosen to represent a range of overlap types and gene functions, and high antisense expression. Both NCU04182 (splicing factor 3 b subunit 4) and NCU07268 (a hypothetical protein with a PAS domain) are associated with convergently-transcribed antisense transcripts. The antisense transcript partially complementary to NCU07268 is significantly up-regulated on exposure to light (Figure 4; RNASeq, *p*_adj_ 6.54×10^−9^; qPCR, *p*-value 0.0084). This antisense transcript is located close to and is expressed divergently from the *blue light-induced-3* gene (*bli-3*, NCU07267). We therefore suggest that *bli-3* and the transcript antisense to NCU07268 may be expressed from a bidirectional promoter. Other predicted sense and antisense transcripts whose expression were verified include the divergently transcribed NCU02607, predicted to code for a hypothetical protein, and its partially complementary antisense transcript. In addition, we confirm the expression of an antisense transcript within which NCU07915 (integral membrane protein) is nested. The antisense transcript overlaps all the introns of the sense transcript and interestingly reads antisense to the third intron of the sense transcript are most abundant. We note that exons 3 and 4 of NCU07915 encode the Mpv17/PMP22 family domain, which is predicted to have pore-forming activity. We also confirmed expression of a known snoRNA that is complementary to the 3′ end of NCU09135 (predicted to code for phosphatidylinositol phospholipase C) and the 5′ end of NCU09136 (predicted to code for a hypothetical protein), and a second antisense lncRNA that is nested within NCU09136 (Figure 4).

Given that the stability of sense/antisense transcript pairs could be controlled by components of the RNA silencing machinery, we assessed the expression of the sense and antisense transcripts by qPCR in a *ddicer (dice-like-1* ^Δ^, *dicer-like-2^RIP^*) knockout in which expression of *dicer-like-1* and *dicer-like-2* is abolished. If sense and antisense transcripts form double-stranded RNA recognized by DICER, we might expect to see higher levels of transcripts in the *ddicer* strain. Another possible consequence of co-expression of sense and antisense transcripts is mis-splicing leading to degradation by the nonsense-mediated decay pathway. NCU04242 is a homolog of *upf1* [42] and is therefore predicted to be involved in the NMD pathway. Increase in the expression of sense/antisense transcripts in the NCU04242 mutant strain would indicate that they are substrates of the NMD pathway (Figure S2). Under the same conditions of light and temperature, we do see that the levels of some of transcripts are significantly different in the *ddicer* strain. The expression of both the sense and antisense transcripts of NCU09135 and NCU09136 was significantly up-regulated in the *ddicer* mutant (*p* value < = 0.05). In some culture conditions we also see up-regulation of the sense transcripts for NCU04182, NCU02607 and NCU07915 and antisense transcripts NCU07268 and NCU02607 in the *ddicer* mutant. With the exception of NCU02607, significant changes in sense and antisense transcript levels are seen for all sense/antisense pairs in the *nmd* mutant.

## Discussion

The presence and functional relevance of widespread transcription in eukaryotic genomes is a subject of extensive debate in the literature. Genome-wide studies have shown that a large portion of the mammalian genome is transcribed while only a small fraction codes for proteins [1], [4]. van Bakel *et al*. [5] argue that most transcription outside of protein-coding regions is accounted for by the presence of reads in introns and on either side of annotated genes that could be a result of alternative promoter usage, alternative exons, and unannotated terminal exons and UTRs. In order to minimise the possibility that our lncRNAs are unannotated extensions of known genes, we focused on transcribed fragments that are distant from annotated genes on the same genomic strand. In total, we report 939 lncRNAs, of which 477 are antisense to annotated genes. Across the whole dataset, the extent of complementary overlap between the protein-coding sense and antisense lncRNAs is large – 371 sense/antisense pairs overlap by more than 500 nts and 236 overlap by more than 1 kb.

### Functions of antisense lncRNAs

The possible modes of action of antisense transcripts are many and varied, and include inhibiting synthesis of their sense transcript, regulating splicing, and controlling the levels of sense RNA via RNAi pathways. It seems likely that features of antisense transcripts, such as the extent and nature of the overlap between sense and antisense transcripts and their expression, may indicate their function. Osato *et al.* [43] have shown in human and mouse that the expression level of transcripts decreases as the region of overlap between the sense and antisense pairs increases. This is consistent with reports that steric clashes of the transcriptional machinery lead to lower expression levels of the transcripts [44]. Here we identify 100 antisense transcripts expressed in our datasets with no accompanying expression of their sense transcripts. This is most often the case when the sense transcript is nested within the antisense, and less common in sense/antisense pairs with 3′ end overlap. This may be due to the absence of specific transcriptional activators of sense transcripts under our growth conditions and/or stronger antisense promoters resulting in steric clashes of the transcription machinery and abortion of all transcription from the sense promoter. Annotated transcripts with dominant antisense expression are enriched for functional categories such as extracellular metabolism, extracellular/secretion proteins, disease and virulence factors.

We find that convergently transcribed sense/antisense pairs overlapping at their 3′ ends predominate, as previously observed in other organisms (see for example [45]). Almost all previously annotated *N. crassa* sense/antisense transcript pairs (97% of those in the BROAD database) also overlap each other at their 3′ ends. A number of signals required for post-transcriptional modifications are located in 3′ UTRs and we might therefore expect that antisense transcripts play roles in regulating modification of their sense counterpart [13]. On average across all *Neurospora* genes, the locations of introns are skewed towards the 5′ end (Figure 1C). Furthermore, our data show that there is an additional significant decrease in the number of introns at the 3′ end for transcripts that have antisense expression. Since most of the sense/antisense pairs overlap at their 3′ ends, interference from antisense transcripts is minimized for splicing of introns at the 5′ end. Antisense transcripts that overlap the introns of protein coding genes, and particularly splicing enhancer signals, have been found to influence alternative splicing of the sense transcript [46], [47]. However, in the light and temperature conditions analysed here we found no evidence of alternative splicing occurring in transcripts with antisense expression.

Some of the NATs that we report here may be required for the production of regulatory siRNAs. Small RNA species, including siRNA, QDE-2-interacting RNA (qiRNA), microRNA-like RNAs (milRNA) and dicer-independent siRNAs, have previously been identified in *N. crassa* [48]. To examine whether our sense/antisense transcripts might form duplexes recognised by DICER-like proteins, we compared the expression of several of our sense/antisense pairs in a *ddicer* mutant strain. Sense and/or antisense transcripts arising from each of the six loci tested were up-regulated in at least one condition of darkness, light or temperature pulse in the *ddicer* strain. However, the sense and antisense transcripts of NCU09135 in temperature-treated samples and NCU09136 in light-pulsed samples were both significantly up-regulated, as might be predicted if they are DICER substrates. It is worth noting that the expression levels of transcripts between experiments in both the *ddicer* and *nmd* strains are relatively large, most likely as a result of the disruption of the associated regulatory pathways, and that we cannot currently distinguish whether the effects of gene deletion on transcript expression are direct or indirect. Recently, small RNAs of 25 nts that do not require DICER for their biogenesis, *dicer*-independent small interfering RNAs (disiRNAs,), have been found associated with regions of convergent transcription and linked to dynamic DNA methylation especially prevalent around promoters of the *disi*-loci [49]. Comparison of small RNA RNAseq data obtained from wild-type and *ddicer* strains should throw light on the presence or absence of disiRNAs mapping to the location of sense/antisense transcript pairs.

### Bidirectional promoters and transcriptional terminators as promoters

Recently, two novel classes of non-coding RNA transcript were annotated in yeast: CUTs (cryptic unstable transcripts) and SUTs (stable unannotated transcripts). CUTs are short (<800 nts) and have a very short half-life in the cell, suggesting that their role is achieved via transcription itself [41], for example, by recruiting histone-modifying enzymes or via transcriptional interference [50]. SUTs, on the other hand are longer, have a longer half-life, and arise from nucleosome-free regions at the 5′ and 3′ ends of actively transcribed genes. Both CUTs and SUTs are found close to the ends of genes suggesting transcription from bidirectional promoters or terminators acting as promoters. The Protein Initiation Complexes (PICs) and Nucleosome Depleted Regions (NDRs), features essential for transcription and found at the 5′ end of protein-coding genes, have also been found to be highly represented at the 3′ end of genes that showed antisense expression [41]. In our dataset, 42% of antisense lncRNAs have start sites between 100 nts upstream and 600 nts downstream of the end of sense transcripts ORFs, and may therefore arise from terminators that also act as promoters. The short half-life of CUTs is attributed to their recognition and degradation by the NMD pathway [51]. Although the novel transcripts we report more closely resemble SUTs, three of the antisense lncRNAs in our datasets showed an increased expression in the *NCU04242* ^Δ^ strain, suggesting that they could be potential substrates for the NMD pathway.

While the majority of lncRNAs in *N. crassa* appear to be transcribed independently of neighbouring genes, we identify examples of potential bidirectional promoters. Seila *et al*. showed that the transcripts formed from bidirectional promoters in mouse are shorter and are less abundant than the sense RNA [52]. In contrast, we find that, of the 355 lncRNAs that have their start sites <1 kb upstream of annotated genes on the opposite strand, 95% of these transcripts were >500 bp in length and there was no evidence that they are less abundant than their sense counterparts on average. Indeed, the transcribed lncRNA is significantly more abundant than the sense transcript in approximately a quarter of the bidirectional pairs. A handful of divergent transcript pairs show a modest positive correlation in their expression in response to light, consistent with their origin from bidirectional promoters.

## Conclusions

Several studies have highlighted the expression of NATs in fungal genomes (reviewed in [19]). We provide the first comprehensive genome-wide study of long non-coding and antisense transcripts expressed in the model filamentous fungus *N. crassa*. The expression profiles of the lncRNAs and antisense transcripts indicate that a variety of mechanisms regulate their expression. However, few examples of antisense transcription have been functionally characterised in any organism. The following questions therefore remain: (1) Which common themes underlie the control of expression of sense and antisense transcripts? (2) Which characteristics of NAT form and expression can be used to predict their mode of action? We suggest that *Neurospora* can serve as an informative model for studying the function of eukaryotic NATs.

## Materials and Methods

### RNA extraction and RNAseq

Cultures of wildtype *N. crassa* (*54-3, bd a*) and *wc-2* ^▵^, *vvd* ^▵^, *frq* ^▵^, *wc-1* ^▵^, *bd* (*quad* ^▵^) strains were grown in liquid medium (1× Vogel's salts, 2% glucose, 50 ng/ml biotin) on a rotary shaker (225 rpm) at 25°C. After 24 hours growth in light and 24 hours in darkness, cultures were either harvested, exposed to a pulse of light (580 μW/cm^2^) for one hour at 25°C, or transferred to 30°C in the dark for one hour (temperature pulse). Cultures (2 biological replicates for each condition – total of 12 samples) were then harvested, ground under liquid nitrogen and RNA extracted (Qiagen). Two rounds of ribosomal RNA depletion were carried out using the RiboMinus kit (Life Technologies). Sequencing libraries were prepared using the Applied Biosystems SOLiD Total RNA-Seq Kit and sequenced using its SOLiD chemistry at the Genomic Technologies Core Facility (University of Manchester, UK). The 50 nt reads were filtered for quality [53] using the following thresholds: minimum count for polyclonal analysis (p = 3), minimum QV for polyclonal analysis (p_QV = 22), maximum errors permitted (e = 10), maximum QV to consider an error (e_QV = 9). The resulting reads were then mapped to the *N. crassa* OR74A (NC10) genome (http://www.broadinstitute.org/annotation/genome/neurospora) [39] using the splice-aware Tophat tool (version 1.4.1) [36]. The mappings were visualised using the Integrative Genomics Viewer (IGV) [54]. Raw sequencing datasets are deposited in the Sequence Read Archive, with accession number SRP035869.

### Annotation of novel transcripts

To annotate novel transcribed regions, all 12 sequencing datasets were merged, and reads that overlap on the same genomic strand were clustered together. Novel transcripts could be made up of several such clusters that do not overlap due to low expression levels, low read coverage [21], or the presence of introns. Analysis of intron size in *N. crassa* revealed a median intron length of 76 nts and 88% of annotated introns are less than 200 nts (Figure S1). Therefore, non-overlapping read clusters closer than 200 nts were further joined together to form larger transcribed fragments (transfrags). Transfrags containing > = 50 reads summed across all WT or quad^▵^ RNAseq datasets were classified as novel transcripts. The genomic locations of transfrags were cross-referenced with the locations of annotated transcripts from the BROAD *N. crassa* genome annotation (NC10) in a strand-dependent fashion. Transcript sequences were searched for coding potential using Coding Potential Calculator [37]. Transcripts with no coding potential were searched against the library of Rfam 11.0 RNA families [55] using INFERNAL 1.1 [56], and for tRNAs using tRNAscan-SE 1.23 [57]. To identify whether any of the remaining transcripts (lncRNAs) sequences are conserved in other fungal genomes, we searched each transfrag against genome sequences of *Neurospora tetrasperma, Magnaporthe grisea, Chaetomium globosum, Myceliophthora thermophile, Trichoderma reesei, Fusarium graminearum, Aspergillus nidulans, Aspergillus oryzae, Aspergillus fumigatus, Neosartorya fischeri*, *Coccidioides posadasii, Myceliophthora thermophila, Thielavia terrestris, Yarrowia lipolytica, Candida albicans, Candida glabrata, Saccharomyces castellii, Saccharomyces cerevisiae and Schizosaccharomyces pombe*, using WU-BLASTN [58], with an e-value cut-off of 10^−5^ and requiring a match covering at least 40% of the length of the query and at least 50 bases.

### Differential expression

Read counts for every gene were calculated from the SAM file [59] using HTSeq (version 0.5.3), and lncRNAs were defined and quantified as above. Read counts for both previously annotated protein coding genes and our lncRNA set were used for the differential expression analysis. The read counts for either WT and light- or WT and temperature-treated samples were normalised and analysed using DESeq [60] at a 5% FDR (adjusted for multiple testing using the Benjamini-Hochberg correction: *p*_adj_-value reported by DESeq). The SAM files were then used to determine alternative splicing in response to light or temperature using Cufflinks [61].

### RT PCR

We validated the expression of 6 sense and antisense transcripts in wildtype, *dicerlike-1* ^Δ^, *dicerlike-2^RIP^* (*ddicer*, in which expression of *dicerlike-1* and *dicerlike-2* is abolished), and *NCU04242* ^Δ^ (deletion of a homologue of *upf1* from *Arabidopsis*) strains by qPCR. *Neurospora* was grown under the conditions described above. After 48 hours of light-dark cycle, cultures were either kept at 25°C in the dark, exposed to a pulse of light for one hour at 25°C, or transferred to 30°C in the dark for one hour (temperature pulse). RNA was extracted from the tissues using TRizol. The RNA (100 μg) obtained from the tissues was DNase-treated (New England Biolabs) for 2 hours at 37°C and then the enzyme was heat-inactivated at 75°C for 10 minutes. The RNA was further purified using the RNeasy Plant mini kit (Qiagen). 1 μg of RNA was used to perform strand-specific reverse transcription using the primers shown in Table S7. Reverse transcription was performed using the RevertAid Reverse Transcriptase kit (Thermo Scientific) using the conditions suggested by the manufacturer, followed by RNase treatment. Quantitative real-time PCR was performed on the cDNA using the custom Taqman gene expression assays (Life Technologies). The TaqMan probes were designed to target the region of overlap between the sense and antisense transcript (see Table S7 for primers and probe sequences). The use of strand-specific RT and the same probe to detect both sense and antisense enables us to confirm the presence of sense and antisense transcripts independently. cDNA was diluted 5 fold and then serially diluted to obtain the standard curve. cDNAs were used at different dilutions based on their expression levels and were quantified using the standard graph. For each sample there were three technical replicates. The cDNA for U2 RNA was used to normalise the expression of the sense/antisense transcript.

## Supporting Information

Figure S1 **A. Distribution of distance between neighbouring annotated transcripts on the same strand. B. Size distribution of introns in annotated transcripts**.
(TIF)

Figure S2 **Expression of both the sense and antisense transcript for NCU04182, NCU07268, NCU02607, NCU07915, NCU09135 and NCU09136 in the WT,** ***ddicer*** **and** ***NCU04242*** **^Δ^ (nmd) strains are shown, after growth in the dark, and exposure to light and temperature pulses.** Black bars indicate the protein-coding sense transcript and white bars indicate its antisense transcript. Each experiment was repeated with 3 biological replicates. Error bars represent standard deviation. Statistical significance between WT and mutants was determined using Student t test, * indicates *p*-value <0.05 and ** indicates *p*-value < = 0.005 (3 biological replicates, each with 3 technical replicates). Only significant differences between the mutant and WT strains are shown here.
(TIFF)

Table S1 **The number of reads obtained from RNA sequencing and mapped to unique locations in the NC10 version of the genome for each sample in the WT and** ***quad^▵^*** **dataset.**
(XLSX)

Table S2 **List of putative lncRNAs and novel putative protein-coding transcripts.**
(XLSX)

Table S3 **List of previously annotated sense/antisense pairs.**
(XLSX)

Table S4 **List of antisense lncRNAs.**
(XLSX)

Table S5 **FunCat analysis.**
(XLSX)

Table S6 **List of lncRNA/lncRNA antisense overlaps.**
(XLSX)

Table S7 **Primers used for strand-specific RT and qPCR.**
(XLSX)
