# Supplementary figures and images for: Natural Antisense Transcripts and Long Non-Coding RNA in Neurospora crassa
Source: PLoS One. 2014 Mar 12;9(3):e91353. doi: 10.1371/journal.pone.0091353 (PMC3951366; doi:10.1371/journal.pone.0091353)

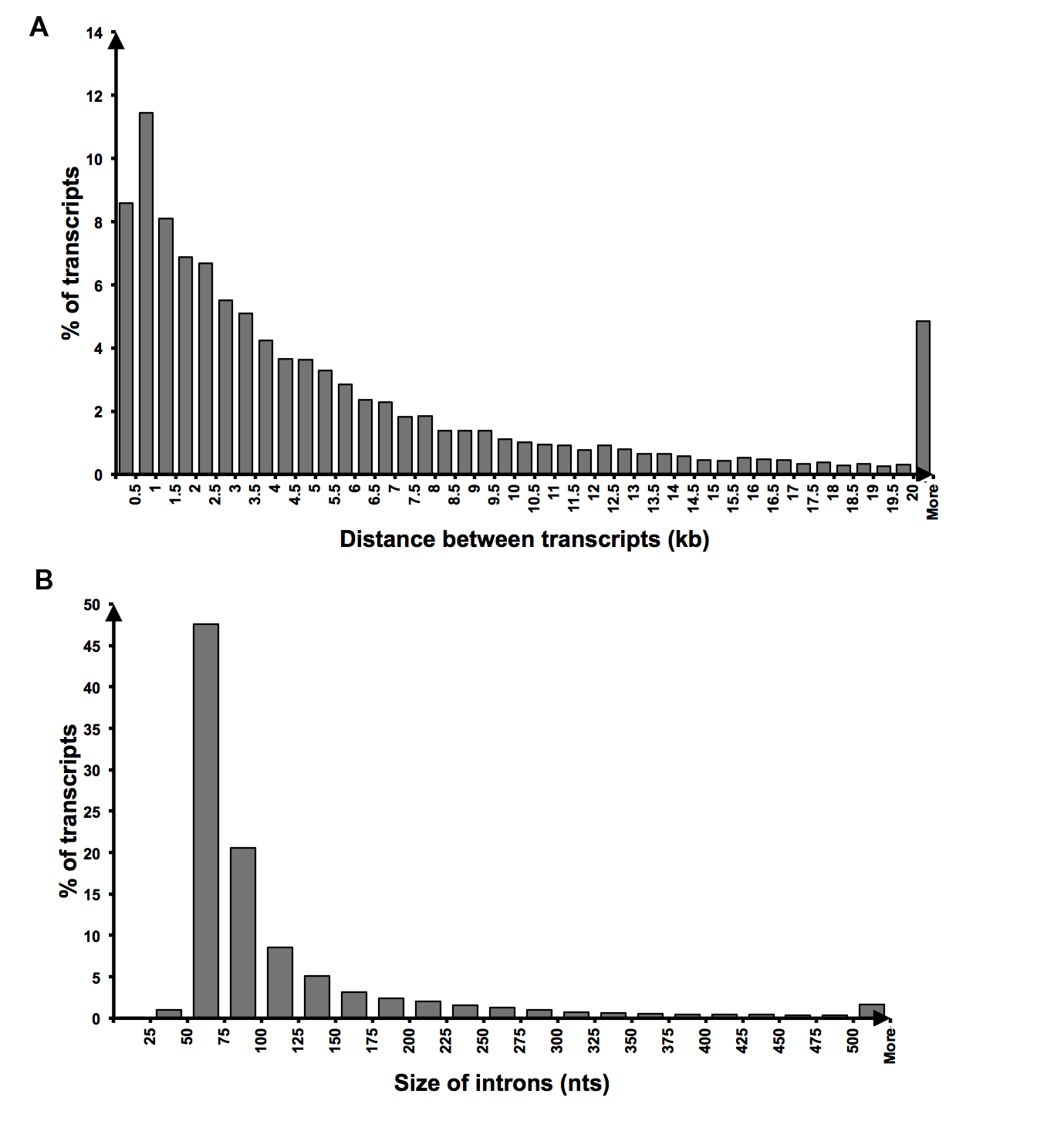

Supplement: Figure S1 — A. Distribution of distance between neighbouring annotated transcripts on the same strand. B. Size distribution of introns in annotated transcripts. (TIF) [file pone.0091353.s001.tif]

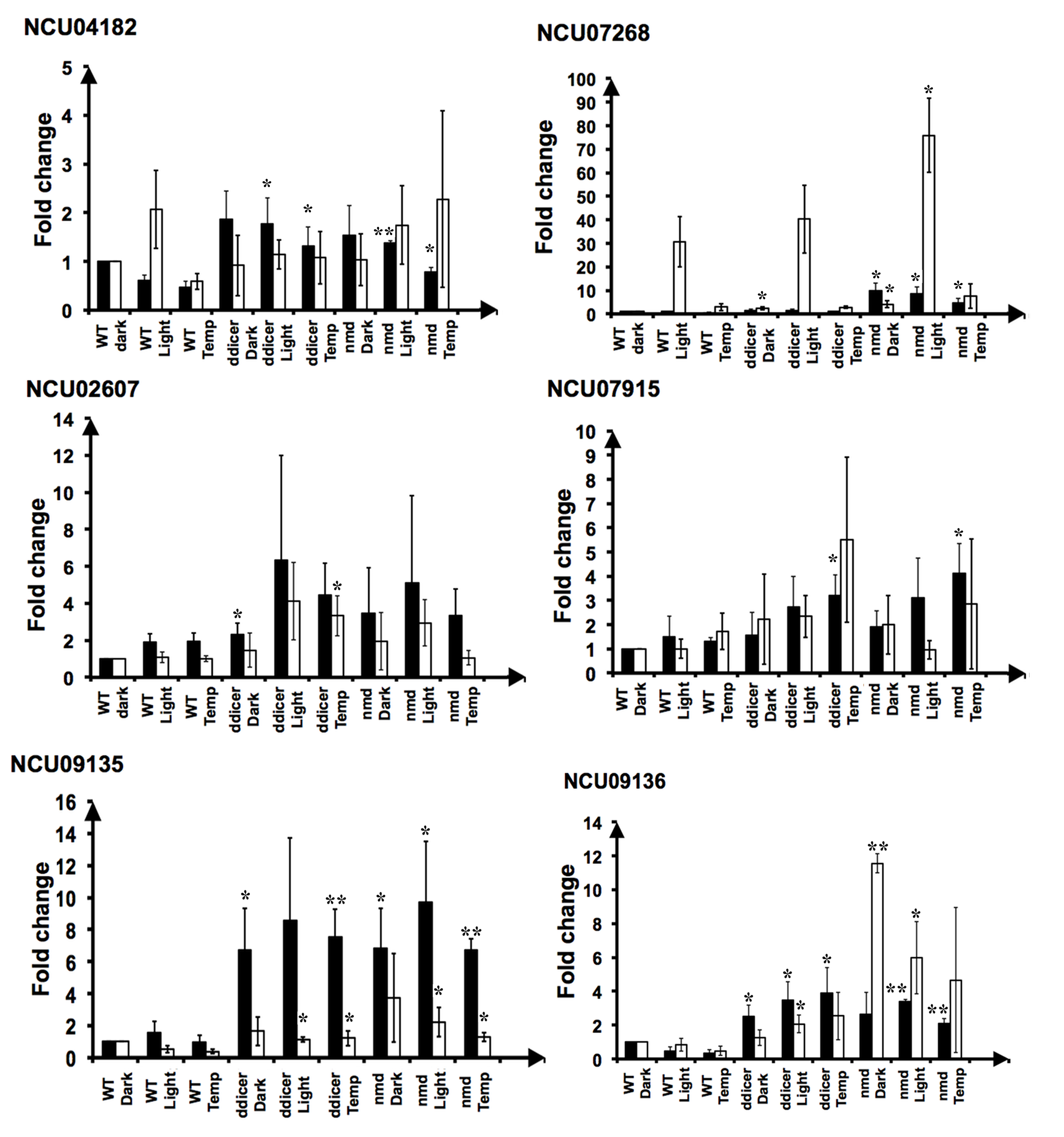

Supplement: Figure S2 — Expression of both the sense and antisense transcript for NCU04182, NCU07268, NCU02607, NCU07915, NCU09135 and NCU09136 in the WT, ddicer and NCU04242 Δ (nmd) strains are shown, after growth in the dark, and exposure to light and temperature pulses. Black bars indicate the protein-coding sense transcript and white bars indicate its antisense transcript. Each experiment was repeated with 3 biological replicates. Error bars represent standard deviation. Statistical significance between WT and mutants was determined using Student t test, * indicates p-value <0.05 and ** indicates p-value < = 0.005 (3 biological replicates, each with 3 technical replicates). Only significant differences between the mutant and WT strains are shown here. (TIFF) [file pone.0091353.s002.tif]
